# Supplementary material for: Bacterial multispecies interaction mechanisms dictate biogeographic arrangement between the oral commensals Corynebacterium matruchotii and Streptococcus mitis
Source: mSystems. 2023 Aug 23;8(5):e00115-23. doi: 10.1128/msystems.00115-23 (PMC10654079; doi:10.1128/msystems.00115-23)
Supplement: Supplemental material — Supplemental results and materials and methods. [file msystems.00115-23-s0001.pdf]

Supporting Information for

Bacterial multispecies interaction mechanisms dictate biogeographical arrangement between the oral commensals *Corynebacterium matruchotii* and *Streptococcus mitis*

Authors: [1]Eric Almeida*, [2]Surendra Puri*, [1]Alex Labossiere, [2]Subhashini Elangovan, [2]Jiyeon Kim, [1]Matthew Ramsey**

***** indicates authors contributed equally

Institutional Affiliations: [1]The University of Rhode Island, Department of Cell and Molecular Biology, [2]The University of Rhode Island, Department of Chemistry, Kingston RI 02881.

****** Corresponding author: 120 Flagg Rd. Kingston, RI 02881, 401-874-9505

Email: mramsey@uri.edu

This PDF file includes:

Supporting text
Figures S1 to S3
Tables S1 to S2
SI References

Supporting Information Text

Supplemental Methods

Growth curves. Five milliliter cultures of *C. matruchoti*, *lutA* deletion, *lutABC* deletion, *lutABC* deletion strain with empty vector, and the complemented *lutABC* deletion were grown in BHI-YE for ~48hr and washed once by centrifugation at 10,000xg , resuspended in and diluted in modified RPMI media supplemented with 20mM lactate at an OD_{600} of 0.025. Cultures were incubated statically at 37°C with 5% CO_2. Optical density readings at 600nm were taken every 4-6 h over a ~120 h period.

Note. Additional SECM methods including tip design, development and acquisition parameters are part of a companion manuscript [1]. Methods below highlight the most relevant aspects of SECM utilized to quantify coculture metabolite exchange of lactate between *S. mitis* and *C. matruchotii*.

Scanning electrochemical microscopy sample preparation. Bacteria were grown overnight in BHI-YE and then washed by centrifugation in defined medium. The defined medium used was an amended version of Teknova EZ RICH (Teknova, M2105). We prepared the medium as described in the manufacturers instructions with the addition of vitamin solution, lipoic acid, folic acid, riboflavin, NAD+ and nucleotides to final concentrations from that of an oral complete defined medium previously described [2] and glucose at 10 mM. Bacteria were grown to an OD600 of 0.3-0.5 and then diluted to final concentrations of $1.2x10^7$ and $6.0x10^6$ CFU of *C. matruchotii* and *S. mitis* respectively in 200 µL of defined medium. This was then incubated at 37°C with 5% CO_2 for 1 h. 10 µL of this solution was then added to a poly-lysine coated glass slide and incubated at 37°C for 15 minutes after which medium was removed by micropipette to remove planktonic cells and ensure only attached cells remained. An additional 10 uL of pre-warmed defined medium was then added to the slide. Samples were then transferred to the SECM instrument for further analysis.

Solution preparation for SECM measurements

Tetraethylammonium chloride (TEA^+Cl^-, Sigma Aldrich) solution of 1mM concentration was made in freshly prepared oral bacterial growth media of ~pH 7.5, where TEA^+ was used as a probe ion. The solution was then filtered with a syringe filter (0.1 µm filter unit, SLVV033RS, Duropore PVDF membrane, MILLEX VV). The SECM cell made with Teflon and glass was cleaned in piranha solution, and thoroughly rinsed with nanopure water (18.2 MΩ·cm, TOC 2 ppb; Milli-Q Integral 5 system, MIllipore).

NanoSECM measurements

Here, we used our home-built nanoSECM for measuring approach curves and imaging at single bacterial cells. Before approaching a submicrosized pipet tip to the bacteria, we measured cyclic voltammograms with tetraethylammonium (TEA^+, Sigma Aldrich) ion and estimate a steady state current in bulk solution ($i_{T,\infty}$) to confirm that nanopipet is working with a reasonable size. Then, our pipet was held c.a. 100 − 200 µm above the glass substrate with the help of a video microscope (Caltex VZM 400 lens with INFINITY2-1R 1.4 Megapixel USB 2.0 Microscopy Camera CCD) and a lockable micropositioner (DM-

25L, Newport). We approached our pipet tip to the bacterial sample with z -piezo by measuring the steady-state current as a function of the distance between a pipet tip and glass substrate, and recorded an SECM approach curve. When the z-piezo moved 50 µm (maximum travel distance for piezo, P-620.ZCL, PI (Physik Instrumente), German) down at 50 nm/s, it was completely withdrawn and manually approached 50 µm with a lockable micropositioner. Then, the pipet tip was approached with z- piezo until it showed a sharp decrease in current, i.e., feedback current response. Once we observed a foot of feedback current response, the pipet tip was withdrawn 6 µm above, and approached at a slower rate i.e., 10 nm/s (1 nm step size with 100 ms incremental time) until our steady-state current decreased to c.a. 93 − 90 % of the $i_{T,\infty}$, which corresponds to a distance of c.a. 2 times of tip radius from the substrate. Subsequently, a tip was further withdrawn 2 µm considering a height of bacteria. Here we approached only 93% of $i_{T,\infty}$ to prevent the crash with possible clumps of bacteria in coculture. Here, SECM imaging was done at the constant height mode. A pipet tip was raster scanned along *x*- and *y*-axis at 1 µm/s (i.e., 100 nm/100 ms) in the region of 25 µm × 25 µm to get a topographical image of the bacteria. After topographical imaging of the bacteria with respect to TEA^+, a pipet tip was brought to the original starting point. We again withdrew z-piezo 6 µm above, approached again at 10 nm/s (i.e., 1 nm/100 ms) until we reached c.a. 90 % of $i_{T,\infty}$, and withdrew 2 µm just right before imaging similar to previous topographic imaging step. A pipet tip potential is switched to a potential to sense lactate ion transfer (0.46 V more positive than diffusion-limiting potential of TEA^+ ion transfer). Continuously, a tip was raster scanned in the same region of 25 µm × 25 µm to real time monitor lactate production/consumption by bacteria in situ.

Electrochemical detection of TEA^+ and Lactate ion transfer by a submicropipet-supported interface between two immiscible electrolyte solutions (ITIES)

To investigate lactate production and consumption by bacteria as well as the topography of bacterial cells, a submicropipet-supported ITIES was employed. With this submicrotip, an etched Ni/Cu electrode in the internal organic electrolyte exerts a bias across the submicroscale liquid/liquid interface against an electrode in the aqueous solution (Fig. S3A and B) to yield the amperometric tip current based on the selective interfacial transfer of a small probe ion (2). A quartz submicropipet is filled with the electrolyte solution of 1,2-dichloroethane (DCE) to detect tetraethylammonium (TEA^+) as a probe ion, and lactate as a metabolite ion produced or consumed by bacteria. Using this pipet electrode, TEA^+ ion transfer (IT) is induced by applying negative potential, which can probe topography of bacterial sample, while lactate is sensed by switching a potential to positive to *in situ* monitor the chemical interaction between bacteria. The coculture of *C. matruchotii* and *S. mitis* is immobilized over a poly L-lysine coated slide glass plate, and studied by scanning or approaching a 800 nm-diameter pipet tip over the bacteria (Fig. S3C and D). More detailed information about the electrochemical sensing of each ion species at the selective interfacial potentials should be found in the companion manuscript [1]. Notably, a submicrometer-sized pipet tip enables the real time study of chemical interactions between bacteria as well as their topography at a single cell level.

Supplemental Results

Quantitative analysis of the permeability of bacterial membranes, and local lactate concentration produced by *S. mitis* and consumed by *C. matruchotii*

After obtaining SECM images, SECM approach curves were measured directly above *S. mitis* and *C. matruchotii*. The tip approached from the bulk solution while recording the steady-state tip current response for TEA^+ IT until contacting the surface of bacterial cell. This resulted in decrease in current as a function of normalized distance to the tip radius (*d/a*) with a sudden spike as a sign of contact between the tip and bacterial membrane (black and grey solid curves in Fig. S3E). The intrinsic permeability of both *S. mitis* and *C. matruchotii* membrane to TEA^+ (k = 2.4 (± 0.1) × 10^{-4} cm/s) was determined by fitting experimental approach curves with a finite element simulation of a two phase SECM problem as described in detail elsewhere (black open circles in Fig. S3E) (2,3). TEA^+ was used in lieu of lactate for determining permeability for two reasons i.e., the same charge amount (± 1) and similar diffusion coefficients of ~6 × 10^{-6} cm^2/s. The approach curves over bacteria are nearly identical to the negative feedback approach curves obtained over an insulating glass substrate (black and grey solid curves in Fig. S3E), indicating that both bacterial membranes are almost impermeable to TEA^+.

With a determined permeability, k of *C. matruchotii* and *S. mitis* membranes, an SECM approach curve over *S. mitis* was further simulated using a two phase SECM problem as well, where tip current responses for lactate IT become higher over *S. mitis* due to *in situ* generation of lactate. Herein, enhanced tip currents to 167 % of $i_{T,\infty}$ for lactate IT obtained at 1.20 d/a during SECM imaging (in Fig. 5C) are used as a criterion to verify the theoretically simulated approach curve. The resulting approach curve is depicted in Fig. S3E, where tip currents increase as the tip approaches over *S. mitis*, and reach 1.66 fold of $i_{T,\infty}$ at 1.2 *d/a* (red open circles in Fig. S3E). The corresponding concentration profile of lactate near *S. mitis* and a pipet tip is shown in Fig. S3F, where the concentration of lactate produced by *S. mitis* is estimated as 0.50 mM very near the bacterial surface. This localized concentration of lactate is two times higher than that in bulk solutions resulting from the diffusion of lactate produced by other ensemble of *S. mitis* cells.

Notably, in approach curves over *C. matruchotii* (blue open circles in Fig. S3E), 48 % of $i_{T,\infty}$ at 1.20 *d/a* is consistent with tip current responses over *C. matruchotii* during constant-height SECM imaging based on lactate IT (~48 % current responses of $i_{T,\infty}$ over *C. matruchotii* in Fig. 5C), which is much smaller than 85 % current responses of $i_{T,\infty}$ based on simple-topographical response (black open circles in Fig. S3E). This result indicates that *C. matruchotii* efficiently depletes localized lactate produced by *S. mitis* in its proximity, thus tip current responses above *C. matruchotii* are not only affected by its intrinsic membrane permeability but also controlled by its characteristic consumption rate, ≥ 5 × 10^6 s^{-1}. Accordingly, tip current response based on lactate IT is lowered as the tip moves laterally toward *C. matruchotii*. More detailed information about the theoretical simulation and the quantitative analysis should be found in the companion manuscript [1].

In summary, this work demonstrates the high significance and power of SECM, which enabled us not only to *in situ* monitor the chemical communication between two commensal bacteria, *S. mitis* and *C. matruchotii* by imaging, but also to quantify the lactate production by *S. mitis* and consumption by *C. matruchotii* in real time at submicron resolution.

183

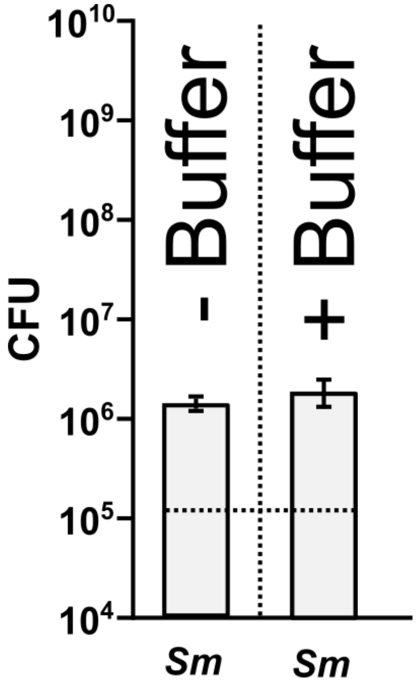

Fig. S1.

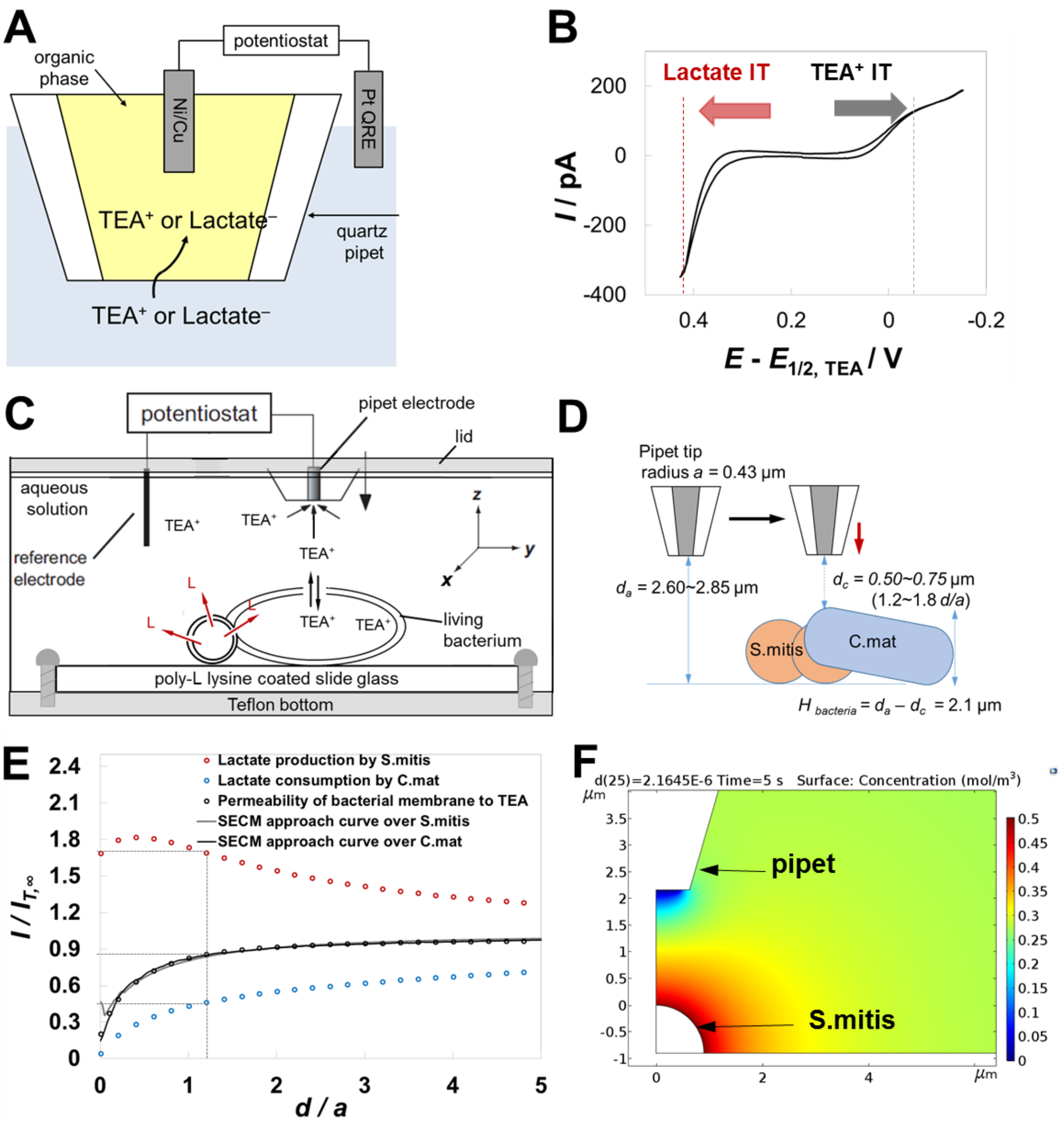
A
organic phase
potentiostat
Ni/Cu
Pt QRE
TEA⁺ or Lactate⁻
quartz pipet
TEA⁺ or Lactate⁻
B
Lactate IT
TEA⁺ IT
200
0
-200
-400
0.4
0.2
0
-0.2
I / pA
E - E1/2, TEA / V
C
potentiostat
pipet electrode
lid
aqueous solution
TEA⁺
TEA⁺
TEA⁺
reference electrode
z
y
x
L
L
L
TEA⁺
TEA⁺
living bacterium
poly-L lysine coated slide glass
Teflon bottom
D
Pipet tip radius a = 0.43 μm
da = 2.60~2.85 μm
dc = 0.50~0.75 μm (1.2~1.8 d/a)
S.mitis
C.mat
H bacteria = da – dc = 2.1 μm
E
Lactate production by S.mitis
Lactate consumption by C.mat
Permeability of bacterial membrane to TEA
SECM approach curve over S.mitis
SECM approach curve over C.mat
I / IT,∞
d / a
F
d(25)=2.1645E-6 Time=5 s Surface: Concentration (mol/m3)
pipet
S.mitis
μm

Figure S2.

Table S1. Lists of plasmids and strains used in study.

**Plasmids**	**Identifier**	**Host**	**Description**
pEAKO2	MR257	DH5-alpha	Markerless clean deletion vector with sucrose counter-selection originated from pMRKO
pEA910	MR317	DH5-alpha	*lutABC* clean deletion vector utilizing pEAKO2 as parent vector
pEA400	MR409	DH5-alpha	*ftn* clean deletion vector utilizing pEAKO2 as parent vector
pEAKO-lutA	MR258	DH5-alpha	*lutA* insertional inactivating kanamycin marker vector utilizing pEAKO2 as parent vector
pAL618	MR543	DH5-alpha	*lutABC* and native promoter region inserted into the *Corynebacterium* shuttle vector pCGL0243 for *in trans* complementation of *lutABC*.
**Species**	**Identifier**	**Strain**	**Description**
C. matruchotii	MR127	ATCC14266	Wildtype strain
C. matruchotii	MR405	*ΔlutABC*	*lutABC* operon clean deletion strain
C. matruchotii	MR414	*Δftn*	*ftn* clean deletion strain
C. matruchotii	MR264	*lutA-kanR*	*lutA* kanamycin-cassette insertional inactivation strain
C. matruchotii	MR546	*ΔlutABC* + EV	MR405 containing the empty *Corynebacterium* shuttle vector pCGL0243
C. matruchotii	MR545	*ΔlutABC* + *lutABC*	MR405 containing the *lutABC* complement vector pAL618 from MR543
S. mitis	MR181	ATCC49456	Wildtype strain
S. mitis	MR291	*ΔspxB*	*spxB* deletion strain

Table S2. *C. matruchotii* differentially expressed genes when in coculture with *S. mitis* aerobically.

Fold change	q value	Gene ID	Gene Name
6.77	0.0009	fig|43768.439.peg.811	FIG00544752: hypothetical protein
4.37	0.003	fig|43768.439.peg.1549	LutA Predicted L-lactate dehydrogenase Fe-S oxidoreductase subunit YkgE
3.76	0.0002	fig|43768.439.peg.1550	LutB Predicted L-lactate dehydrogenase Iron-sulfur cluster-binding subunit YkgF
3.20	0.010	fig|43768.439.peg.1551	LutC Predicted L-lactate dehydrogenase hypothetical protein subunit YkgG
2.96	0.005	fig|43768.439.peg.106	hypothetical protein
2.46	0.0006	fig|43768.439.peg.2696	LSU ribosomal protein L9p
2.39	0.013	fig|43768.439.peg.491	Bacterial non-heme ferritin (EC 1.16.3.2);Ontology_term
2.10	0.004	fig|43768.439.peg.2766	hypothetical protein
-2.02	0.038	fig|43768.439.peg.494	Cytochrome c oxidase polypeptide I (EC 1.9.3.1);Ontology_term
-2.03	0.042	fig|43768.439.peg.1507	ATP synthase epsilon chain (EC 3.6.3.14);Ontology_term
-2.09	0.044	fig|43768.439.peg.2518	Proton/glutamate symporter @ Sodium/glutamate symporter
-2.11	0.044	fig|43768.439.peg.1692	Enolase (EC 4.2.1.11);Ontology_term
-2.11	0.036	fig|43768.439.peg.1089	hypothetical protein
-2.56	0.002	fig|43768.439.peg.1366	PTS system beta-glucoside-specific IIB component / PTS system beta-glucoside-specific IIC component / PTS system beta-glucoside-specific IIA component
-2.65	0.0005	fig|43768.439.peg.936	Site-specific tyrosine recombinase XerC
-2.80	0.001	fig|43768.439.peg.349	Phosphoribosylformylglycinamidine synthase synthetase subunit (EC 6.3.5.3);Ontology_term
-3.00	0.003	fig|43768.439.peg.1196	Iron-sulfur cluster regulator SufR
-4.10	0.0005	fig|43768.439.peg.1202	PaaD-like protein (DUF59) involved in Fe-S cluster assembly
-4.33	0.003	fig|43768.439.peg.1198	Iron-sulfur cluster assembly protein SufD
-6.02	1.24E-08	fig|43768.439.peg.1200	Cysteine desulfurase (EC 2.8.1.7) > SufS;Ontology_term
-6.05	2.49E-05	fig|43768.439.peg.1199	Iron-sulfur cluster assembly ATPase protein SufC

200

SI References

1. Puri, S. R. *et al.* Mechanistic Assessment of Metabolic Interaction between Single Oral Commensal Cells by Scanning Electrochemical Microscopy. *Anal. Chem.* **95**, 8711–8719 (2023).
2. Brown, S. A. & Whiteley, M. A Novel Exclusion Mechanism for Carbon Resource Partitioning in *Aggregatibacter actinomycetemcomitans*. *J. Bacteriol.* **189**, 6407–6414 (2007).
